# Postural control of the trunk in individuals with and without low back pain during unstable sitting: A protocol for a systematic review with an individual participant data meta-analysis

**DOI:** 10.1371/journal.pone.0268381

**Published:** 2022-05-12

**Authors:** Mansour Abdullah Alshehri, Wolbert van den Hoorn, David M. Klyne, Paul W. Hodges

**Affiliations:** 1 NHMRC Centre of Clinical Research Excellence in Spinal Pain, Injury & Health, School of Health and Rehabilitation Sciences, The University of Queensland, St Lucia, QLD, Australia; 2 Physiotherapy Department, Faculty of Applied Medical Sciences, Umm Al-Qura University, Mecca, Saudi Arabia; UNITED STATES

## Abstract

**Introduction:**

Postural control of the trunk is critical for performance of everyday activities and the health of spinal tissues. Although some studies report that individuals with low back pain (LBP) have poorer/compromised postural control than pain-free individuals when sitting on an unstable surface, others do not. Analyses commonly lack the statistical power to evaluate the relevance of features that could impact the performance of postural control, such as sex, age, anthropometrics, pain intensity or disability. This paper outlines a protocol for a systematic review with an individual participant data (IPD) meta-analysis that aims to synthesise the evidence and evaluate differences of postural control measures between individuals with and without LBP during unstable sitting.

**Methods and analysis:**

A systematic review with IPD meta-analysis will be conducted according to PRISMA-IPD guidelines. To identify relevant studies, electronic databases and the reference lists of included articles will be screened. Unstable seat movements are derived from centre of pressure (CoP) data using a force plate or angle of the seat using motion systems/sensors. The comprehensiveness of reporting and methodological quality of included studies will be assessed. Analysis will involve a descriptive analysis to synthesise the findings of all included studies and a quantitative synthesis using two-stage IPD meta-analysis of studies that include both individuals with and without LBP for which IPD set can be obtained from authors. Analyses will include consideration of confounding variables.

**Ethics:**

Exemption from ethical approval was obtained for this review (University of Queensland, ID: 2019003026).

**Systematic review registration:**

PROSPERO ID: CRD42021124658.

## Introduction

Low back pain (LBP) is a complex and multifactorial condition [1]. It is the most prevalent musculoskeletal pain and the leading cause of disability globally [2, 3]. Recurrent episodes of LBP are frequent [1, 4, 5] and some become chronic [6, 7]. One factor that has been argued as a risk for development and perpetuation of the condition is quality of postural control of the trunk, which may underpin mechanics that impact tissue health. However, LBP is a heterogeneous condition and findings from many small studies are inconsistent. This paper proposes a protocol for a systematic review with individual participant data (IPD) meta-analysis that measured postural control in individuals with and without LBP when sitting on an unstable seat.

**Fig 1:**
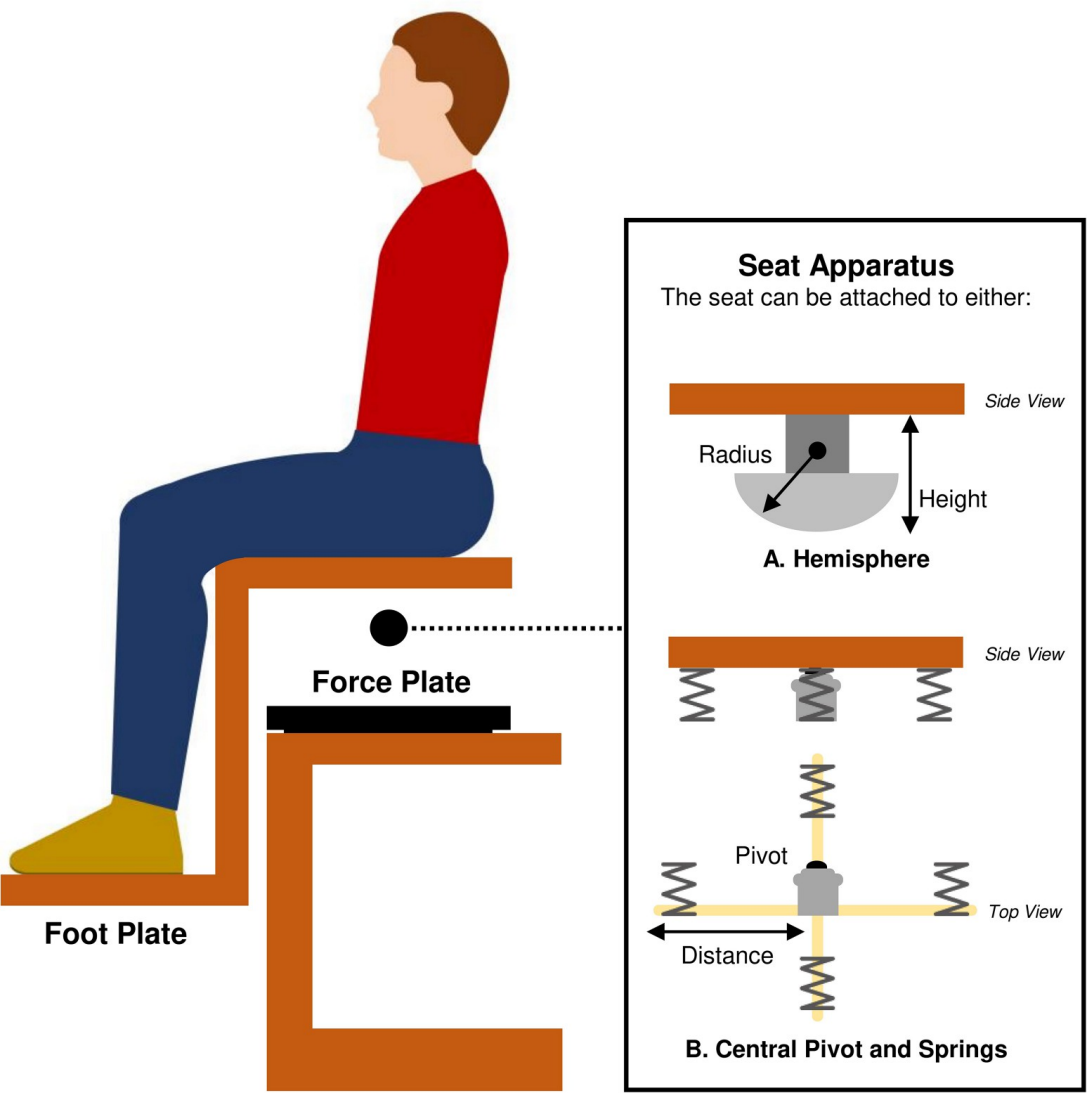
An overview of the unstable sitting paradigm. This paradigm involves sitting on an unstable surface. A seat is attached to (A) a hemisphere or (B) a central pivot with four springs. The task difficulty depends on seat build characterises. Both seats are commonly positioned on a force plate. A foot plate is usually attached to the seat that accommodates the knees at 90° flexion. Maintenance of feet position using the foot plate reduces the contribution of lower limbs to the control of postural equilibrium.

Postural control of the trunk is critical for everyday activities and is integral for the safe execution of human motion [8]. It requires motor skill [9] involving the integration of feedback of position and movement from visual, vestibular and the proprioceptive systems [10, 11] and generation of coordinated motor output using an array of muscles [8, 12, 13]. An unstable sitting paradigm was developed to provide a detailed assessment of postural control of the trunk [14]. This involves sitting on an unstable surface attached to a hemisphere [14] or a chair attached to four springs that moves around a central pivot [15] (Fig 1). Limited contribution from the arms and legs during this balancing task means that equilibrium is maintained primarily by trunk movement [16]. Generally, the seat is positioned on a force plate and its movements are reflected by its centre of pressure (CoP)–the location of the point of contact of the seat hemisphere [14, 16] or the barycentre of the forces under the wobble chair with springs [17]. This unstable sitting paradigm requires the coordinated movement of trunk segments to maintain the body’s centre of mass over the CoP to achieve postural equilibrium [18, 19]. This is generally achieved by movement of the seat to stabilise the upper body [20]. Many outcome measures related to the amplitude of seat motion (e.g., root mean square [RMS] displacement, sway path/mean velocity, range, mean frequency and other measures related to CoP dispersion dynamics [diffusion analysis]) have acceptable to excellent test-retest reliability for assessing postural control in individuals with or without LBP [14, 21, 22]. Commonly, greater CoP movement or seat motion is interpreted as poorer or impaired postural control [12, 16, 23].

Over the last two decades individuals with and without LBP have been studied using unstable sitting paradigms. Some studies suggest postural control of the trunk is compromised in LBP [12, 16, 23–25] and others report no difference [26–28]. These conflicting findings might be explained by the variability of participants’ characteristics (e.g., LBP heterogeneity), experimental setups/procedures (e.g., seat apparatus and its build characteristics) or statistical approaches used; particularly whether important covariates/confounders (e.g., sex, age and body mass index [BMI]) were or were not accounted for in the analysis. Progress of understanding of postural control of the trunk in LBP would be aided by a systematic review of available studies and IPD meta-analysis. IPD meta-analysis enables the calculation of more confident estimates of effects, inclusion of explanatory covariates [29], adjustment for confounding factors [30, 31], and exploration of heterogeneity between studies [30].

Several prior systematic reviews have considered postural control in individuals with and without LBP [21, 32–36]. In general these have included studies that assessed postural control in tasks/movements that involve the whole body, such as standing [21, 32, 34, 36] that can been maintained by adjustments in other body segments such as the lower limbs (e.g., ankles, knees, hips) the spine/trunk, or both [14]. In contrast, postural control in unstable surface depends primarily on the spine (e.g., lumbar spine) [14, 16], which is likely to provide important insight into the mechanisms underlying control of the lumbar region. An expanding number studies of postural control have tested unstable sitting (using similar methodologies) in individuals with and without LBP, but the conclusions are inconsistent and a systematic review and/or meta-analysis of the data is necessary to make progress.

This paper describes a protocol for a systematic review with IPD meta-analysis (when data are available) and narrative review (when IPD are not available) of studies that used an unstable sitting paradigm to investigate postural control in individuals with and without LBP. The aims are to undertake a systematic review with IPD meta-analysis to:

1. Synthesise available evidence regarding postural control of the trunk in individuals with or without LBP when seated on an unstable surface,
2. Summarise the experimental methods of included studies,
3. Assess the comprehensiveness of reporting and methodological quality of included studies,
4. Use IPD meta-analysis to determine whether postural control (as measured by RMS displacement and sway path velocity of CoP/seat) differs between individuals with or without LBP when potential confounders are included (e.g., sex, age, BMI) using studies that included both individuals with and without LBP,
5. Determine whether similar conclusions are derived from alternative outcome measures that are available for fewer studies (e.g., stabilogram diffusion analysis) using IPD meta-analysis,
6. Identify whether postural control outcomes in individuals with LBP depend on pain intensity, pain duration, disability and psychological features,
7. Compare the outcomes of IPD meta-analysis of the studies that included both LBP vs control participants with; (a) the outcomes of studies that tested only LBP or controls using IPD analysis (standardised statistical methods [including covariates]); and (b) narrative review of studies with outcomes for which IPD could not be obtained.

## Methods and analysis

### Design and registration and ethics

This protocol for a systematic review including a descriptive analysis and IPD meta-analysis was planned according to the Preferred Reporting Items for Systematic Review and Meta-Analysis Protocols (PRISMA-P) guidelines [37]. The study has been registered in PROSPERO (registration number: CRD42021124658). Ethical approval was not required for the systematic review and for IPD meta-analysis, ethics exemption from Human Research Ethics Review, The University of Queensland (ID: 2019003026) has been obtained.

### Search strategy

The following electronic databases will be searched from their date of establishment to identify relevant published studies: MEDLINE via EBSCO (1946-), CINAHL via EBSCO (1937-), Embase via Elsevier (1966-), Scopus via Elsevier (1966-) and Web of Science via Clarivate (1900-). The reference lists of all included studies will be screened for any other relevant studies. The corresponding authors of final included studies will be contacted and asked if they have other studies on the same topic. Search terms were determined based on the inclusion criteria (see Table 1). The search in the electronic databases will be limited to; (1) title and abstract, (2) human, (3) adult and (4) English language reports.

**Table 1:** The search strategy and terms.

Step 1	Step 2	Step 3	Step 4
1. low back pain 2. lower back pain 3. back pain 4. LBP 5. CLBP 6. NSLBP 7. low back ache 8. lower back ache 9. back ache 10. backache 11. low back injury 12. lower back injury 13. back injury 14. lumbar pain 15. lumbago 16. healthy 17. pain-free 18. symptom-free 19. without pain 20. subjects 21. participants 22. adults 23. individuals 24. volunteers 25. or (1–24)	26. balance 27. balance control 28. postural balance 29. postural control 30. stability 31. postural stability 32. trunk stability 33. spine stability 34. motor control 35. trunk control 36. spine control 37. postural sway 38. equilibrium 39. kinematics 40. cent* of pressure 41. CoP 42. or (26–41)	43. sit 44. sitting 45. unstable sitting 46. seat 47. unstable seat 48. seated 49. unstable seated 50. chair 51. wobble chair 52. unstable chair 53. or (43–52)	54. and (25, 42, 53)

Searching limiters: (1) title and abstract, (2) human, (3) adult and (4) English language reports.

### Eligibility criteria

#### Inclusion criteria

All cross-sectional studies, clinical trials and cohort studies with baseline data that investigated postural control of the trunk using an unstable sitting paradigm among adult participants aged 18 years or over will be considered. Studies must include any of the following: (1) individuals with LBP and individuals without LBP (healthy controls), (2) individuals with only LBP (3) or studies with only healthy individuals. Participants with any form of LBP will be considered (e.g., acute, subacute or chronic).

Participants must perform postural control tasks using an unstable sitting paradigm that allows movement in all directions/planes (three-dimensional degrees of freedom). Postural control of the trunk should be measured by quantifying seat motion, either quantified as angle of the seat or CoP data generated from a force plate.

#### Exclusion criteria

Studies with insufficient details to determine eligibility will be excluded if the authors do not respond to our requests (at least two attempts via email) to provide the required information. Intervention studies will be excluded, except where baseline data are available. Non-English reports will be also excluded. For individuals with LBP, studies will be excluded if they included participants with major neurological disorders (except for sciatica–pain due to sciatic nerve compression), known/reported major spinal structure deformities (e.g., scoliosis), cancer, infection, or who had undergone a major back surgery in the previous 12 months. For individuals without LBP (e.g., controls), studies will be excluded if they included participants with a major injury or pain, or who had undergone major surgery in the previous 12 months.

Studies will be excluded if they investigated disorders or diseases other than LBP; investigated standing balance or dynamic task conditions such as walking and sit-to-stand; involved different seated balance tasks such as provision of visual feedback or moving the seat to specific target locations; involved sitting on a hemisphere/springs but with feet supported on the floor; involved sitting tasks with perturbations; involved sitting on soft surfaces (such as swiss ball, foam, air cushion); or studies that refer to an already identified dataset (only will be excluded from the quantitative/IPD analysis).

#### Outcome measures

The primary outcome measures are RMS displacement and sway path velocity of the seat during balance trials in which participants balanced with eyes open or closed in the forward-to-backward (anteroposterior) and side-to-side (mediolateral) directions. Greater RMS and sway path velocity are generally considered to reflect poorer balance control. These measures are those most commonly used to quantify postural control. RMS displacement [14, 17] and sway path/mean velocity [14, 17, 22] measures have high test-retest reliability during unstable sitting tasks. These measures of postural control have also successfully differentiated between individuals with and without pathologies [38]. Secondary outcome measures will include those less commonly available, including maximum distance (range), mean power frequency (MPF), and measures related to stabilogram diffusion analysis [14, 17]. Further information about outcome measures including abbreviations, units and descriptions can be viewed in Table 2.

**Table 2:** Postural outcome measures in anteroposterior or mediolateral directions.

Outcome	Unit		Description
	CoP	Angle	
**Primary outcomes**			
RMS displacement	mm	degree (°)	Root mean square displacement of CoP after subtracting the mean position
Sway path velocity	mm/s	°/s	Total absolute path length travelled by CoP per second
**Secondary outcomes**			
Range	mm	°	Distance between minimum and maximum CoP position
MPF	Hz	Hz	Mean power frequency of CoP movements
D	mm^2^/s	°^2^/s	Diffusion coefficient that reflects how fast (slope) CoP is diffusing (spreading). Sometimes referred to as the energy/stochastic activity of CoP motion
- D*s*	mm^2^/s	°^2^/s	Linear slope fitted to the early part of the diffusion-time profile (short-term diffusion coefficient)
- D*l*	mm^2^/s	°^2^/s	Linear slope fitted to the later part of the diffusion-time profile (long-term diffusion coefficient)
CP	-	-	Critical point reflects the intersection coordinates (time and distance) of the short and long-term slopes
- CP*t*	s	s	Mean time coordinate of the critical point
- CP*d*	mm^2^	°^2^	Mean squared distance coordinate of the critical point

CoP, centre of pressure.

### Study selection

Endnote software will be used to collect the search results from all databases and other sources and to remove any duplicates. Article titles and abstracts will be screened for potential inclusion against the defined eligibility criteria by two independent reviewers. Both reviewers are familiar with systematic reviews and meta-analysis studies. For articles that potentially meet eligibility criteria based on titles and abstracts screening, full-texts will be reviewed for final decision. Disagreement between reviewers will be resolved by consensus or a third reviewer. Cohen’s kappa (inter-rater reliability) analysis will be performed to assess the degree of agreement between both reviewers who will make the selection of articles. The number of included and excluded articles will be recorded. Reasons for the exclusion of studies will be recorded.

### IPD collection

The corresponding authors of the included studies will be requested (via email) to share their IPD via email using author information reported in the article (indexed in PubMed database), or official profiles on their affiliated university websites. If no response is received from the corresponding author, co-authors will be contacted. All authors will be informed about IPD meta-analysis and asked if they are willing to provide their IPD set. All authors will receive a summary of proposed study and methods. Only authors of this protocol will have access to the received IPD files. Any eligible studies for which IPD could not be obtained (e.g., authors do not respond or do not have access/authorization to provide IPD set) will be retained for the narrative analysis.

### Data extraction

Study- and individual-level data will be extracted using a standardised form tailored to the requirements of this review. The extracted data will include:

- Study characteristics (author(s), year of publication, study design, setting/lab and country),
- Participant characteristics (e.g., sex, age, height, weight and BMI),
- LBP characteristics/clinical features (pain intensity, disability levels and psychological factors),
- Inclusion and exclusion criteria of the study,
- Experimental setup (seat apparatus [e.g., hemisphere or central pivot with springs, see Fig 1], seat build characteristics [hemisphere diameter and seat height with respect to force plate; springs height, stiffness and positions from the central pivot], presence of a safety rail, presence of an adjustable footplate attached to the seat to support the feet, presence of a force plate, type of recorded data [CoP data or seat angle], sampling rate and data filtering characteristics),
- Experimental protocol (visual balance condition [balancing with eyes open, feedback or eyes closed], duration of the trial, number of trials/repetitions for each visual balance condition, rest time between each trial and task, randomisation used, instructions given to participants [e.g., “sit as still as possible”], arms position, task difficulty [usually based on seat apparatus and seat build characteristics], number of participants touching the safety rail and whether these trial were excluded),
- Any reported adverse effects during the assessment of postural control,
- Postural outcome measures (see Table 2),
- Primary reported findings and conclusions.

### IPD preparation

All datasets will be stored in a master spreadsheet and will be carefully screened in terms of presentation of overall data and available baseline variables, and the unit of measurement for each outcome measure will be unified. If any important data are not available/provided, the primary author will be contacted.

### Covariates

Balance performance when sitting on an unstable seat is potentially influenced by characteristics of the participants [14, 27] and the experimental setup [14, 27]. These characteristics can be divided into three main categories: (1) individual factors (e.g., age, height, weight, sex) [14, 27]; (2) in the case of LBP, clinical factors such as pain intensity and disability levels which could potentially impact balance performance; and (3) experimental factors such as the difficulty level of the seat apparatus (e.g., seat attached to hemisphere or central pivot with springs) [14, 27]. Assessment of potential and commonly reported confounders/covariates will be made and used for further statistical analysis (see statistical analysis below). This approach will provide highest available statistical power by avoiding exclusion of studies (and/or participants) that did not report all potential covariates.

### Comprehensiveness of reporting and methodological quality

There is currently no available scale to assess the comprehensiveness of reporting and methodological quality of studies of postural control. Further, available validated checklists such as the STROBE checklist do not include items asking specific information about the characteristics of LBP or that consider components related to the experimental setup (e.g., seat build characteristics) and protocol (e.g., trial duration and task repetition) of postural control tasks. To account for this, we adapted a checklist to assess the comprehensiveness of reporting and the methodological quality of features that we considered to be critical. This checklist includes components from quality checklist developed by a previous systematic review on postural control [34] and recommendations regarding standardized CoP methods reported by another systematic review [21].

The quality checklist has 25 items with a ‘yes’, ‘partially’ and ‘no’ option to assess the comprehensiveness of reporting and methodological quality of studies across five main domains including: participant characteristics, LBP characteristics, experimental setup/protocol, confounding effects control, and statistical information. The content and description of the checklist is explained in Table 3. Each item will be scored as ‘1’ if answered ‘yes’, ‘0.5’ if answered ‘partially’ (some information are provided) and ‘0’ if answered ‘no’. The overall quality score is the sum of all scores converted to a percentage. A separate quality score will be calculated for each domain. Quality scores range from 0 to 100%, with higher scores indicating higher quality. Two independent reviewers will assess the comprehensiveness of reporting and the methodological quality of all included studies. Discrepancies between both reviewers will be settled by consensus and a third reviewer will be involved when necessary.

**Table 3:** A checklist for comprehensiveness of reporting and quality of the methods.

Domain	Item	Description of information that should be provided (R: report) or methodological issues that influence the quality of interpretation (Q: quality)	Available in published paper (Y/P/N)	Available from author for IPD (Y/P/N)
Participants’ characteristics	Age	R: Summary measure (e.g., range or mean and SD) [34]		
	Sex	R: Number or proportion of male/female [34]		
	Height	R: Summary measure (e.g., range or mean and SD) [34]		
	Weight	R: Summary measure (e.g., range or mean and SD) [34]		
	Specific participant group	R: Information about whether or not the participants are from a specific participant group (e.g., participants of a specific sport; military; ethnicity; etc.)		
	History of LBP	R: Information regarding whether controls/healthy participants had history of LBP		
LBP characteristics	Type	R: Type of LBP (e.g., non-specific, stenosis, etc.)		
	Symptom duration	R: Information about duration of LBP [34] to determine whether pain is: acute (0–6 weeks), subacute (7–12 weeks) or chronic (>12 weeks) [39]		
	Severity level	R: Pain intensity level [34] using a valid and reliable scale (e.g., VAS and NPRS)		
	Disability level	R: Disability level [34] using a valid and reliable scale (e.g., ODI and RDQ)		
	Psychological factors	R: Psychological factors identified using a valid and reliable scale (e.g., FABQ and PCS)		
	Seat apparatus	R: Information about the seat build characteristics		
Experimental setup/protocol	Visual balance condition	R: Information about the visual balance condition (e.g., eyes open and/or eyes closed)		
	Trial duration	Q: A minimum duration of 30 seconds for each trial [21]		
	Repetition	Q: At least three repetitions [21]		
	Instructions	R: Instructions given to participants before recording [40]		
	Sampling & filtering	R: Information about the sampling rate and applied low pass filter characteristics [21]		
	Outcome measures	R: Clear description about how outcome measures were calculated (e.g., descriptions, equations or a citation of article)		
	Excluded data	R: Information about excluded participants/trials		
Confounding effects control	Age	Q: Adjustment for age in statistical analysis [34]		
	Sex	Q: Adjustment for sex in statistical analysis [34]		
	Height	Q: Adjustment for height in statistical analysis [34]		
	Weight	Q: Adjustment for weight in statistical analysis [34]		
Statistical information	Statistical method	R: Adequate information about the statistical methods used for analysis [34]		
	Sample size	R: Information about power calculation		

Y, yes; P, partially; N, no; IPD, individual participant data; SD, standard deviation; LBP, low back pain; VAS, Visual Analogue Scale; NPRS, Numeric Pain Rating Scale; ODI, Oswestry Disability Index; RDQ, Rolland-Morris Disability Questionnaire; PCS, Pain Catastrophising Score; FABQ, Fear-Avoidance Beliefs Questionnaire.

### Descriptive analysis

To address Aims 1–3, a descriptive analysis will be used to synthesise current evidence regarding postural control of the trunk in individuals with or without LBP when seated on an unstable surface, and summarise main characteristics of all eligible studies (see Table 4). All studies will be included, including those that only report data for LBP or healthy controls, and studies for which data are unavailable, and thus not included in the IPD meta-analysis. The comprehensiveness of reporting and methodological quality of included studies will be reported. These data will be presented in the form of tables and/or figures as appropriate.

**Table 4:** List of data for the descriptive analysis.

Characteristics	Experimental setup	Experimental protocol	Results
First Author Year of publication Sample size *All participants*: Sex Age Height Weight BMI *LBP only*: Pain type Pain form Pain duration Pain intensity Disability level Psychological factors	Seat apparatus Seat build characteristics Presence of a foot plate Presence of a safety rail Presence of a force plate Type of recoded data	Position of arms Visual balance condition Task duration Number of repetitions Given instructions	*Main findings (e*.*g*.*)*: Differences in postural control measures between LBP and controls Differences in postural control measures between visual balance conditions or task difficulty levels Reliability of CoP parameters during unstable sitting tasks

BMI, body mass index; LBP, low back pain; CoP, centre of pressure; SD, standard deviation; SE, standard error; CI, confidence interval; IQR, interquartile range.

### IPD meta-analysis

To address Aims 4–5, a quantitative analysis will be conducted using two-stage IPD meta-analysis to investigate postural control of the trunk in individuals with and without LBP when sitting on an unstable surface, while considering the characteristics of each individual participant. The two-stage IPD meta-analysis will be obtained by (1) analysing IPD from each study separately to calculate aggregate data of interest using multilevel mixed-effects models, then (2) combining the results using conventional meta-analysis methods. The advantage of this approach is that it applies a standardised statistical method and control of covariates to all datasets [41, 42]. Data will be used to generate forest and funnel plots, and to investigate between-study heterogeneity. The analysis will be limited to only studies that include both individuals with LBP and those without LBP (controls) to identify between-group differences in postural control and will be performed for each identified outcome and visual balance condition (eyes open and eyes closed) and direction (anteroposterior and mediolateral directions). A separate analysis will be considered to investigate the interaction between group (LBP and control) and main covariates (sex, age and BMI), and between group and visual balance condition if applicable.

The IPD meta-analysis will account for the clustering of participants within studies using random-effect models to avoid misleading effect estimates and potentially inappropriate conclusions [43]. A random effect with restricted maximum likelihood (REML) method estimation (when the heterogeneity is small and when study sizes differ substantially) or Hartung and Knapp method estimation (when the heterogeneity is large and when study sizes are similar) [44] will be used.

Overall and individual study effect sizes will be estimated. Mean difference (MD) will be used if the outcome is measured in the same unit for all studies. Standardised mean difference (SMD) will be selected if the outcome is measured in different units across studies [45]. I^2^ index (degree of heterogeneity) will be reported which can be classified into four categories [46]: (1) non important heterogeneity (I^2^ = 0–40%), (2) moderate heterogeneity, (I^2^ = 30–60%), (3) substantial heterogeneity (I^2^ = 50–90%), and (4) considerable heterogeneity (I^2^ = 75–100%).

The result will be regarded as statistically significant if *p<*0.05. Analyses will be performed using Stata/IC 16.1 software (Release 16, StataCorp LLC, College Station, Texas, USA). The main results will be presented in tables or figures as appropriate.

To address Aim 6, a two‐stage IPD analysis will be performed on data from studies with LBP participants to identify the relation between clinical features (e.g., pain, disability or fear-avoidance) and postural control measures using multilevel mixed-effects models.

To address Aim 7, descriptive statistics (e.g., means and standard errors) of postural outcome measures in studies with only healthy or LBP individuals will be plotted with data from other studies with both groups (LBP versus controls) using IPD (if available) or aggregate data, by application of standardised statistical methods (multilevel mixed-effects models) including covariates. Outcomes from studies for which IPD could not be obtained will be summarised briefly in a narrative manner and contrasted with the findings of the IPD meta-analysis.

### IPD group collaboration and publication policy

The IPD meta-analysis project is undertaken by two teams: the primary team and the collaborative team. The primary team (authors of this protocol paper) will lead the project, perform the initial screening of literature, and identify the eligibility criteria. They will be responsible for management of the study progression, organizing interactions and meeting discussions with the collaborative group on a regular basis. The collaborative team will include a primary/representative author from each included dataset. New collaborators will be invited to this study as new eligible studies are identified.

The IPD analysis and the manuscript will be prepared and written by the primary team. All researchers from both teams will approve the final version of the manuscript for publication. Collaborative team members will be invited to be recognised as authors or by acknowledgment depending on their involvement (a significant intellectual or scholarly contribution to the study as per recommendations for authorship by Australian Code for the Responsible Conduct of Research) [47] and preference. The data will not be utilised for any objectives not already defined without the permission of contributors and primary authors of the original studies.

### Ethical considerations

All data collected have been deidentified and are from participants who provided informed consent to the original authors. However, each author from the included IPD studies will be sent a ‘Memorandum of Understanding’ to sign, containing a summary of this study followed by items regarding the ownership and confidentiality of their data. Authors will be invited to disclose any restrictions or terms on how to use or store their data that they wish to apply. They will also be asked to agree to provide access to their raw dataset, and state their preference for engagement in this IPD meta-analysis (e.g., co-authorship). The access to all received de-identified data will be limited to only the members of the primary team.

## Discussion

This paper describes the protocol to perform a systematic review with an IPD meta-analysis to assess the postural control of the trunk in individuals with and without LBP during unstable sitting and whether there are differences in primary and/or secondary postural outcome measures. The statistical methods that will be applied will facilitate exploration of the impact of participant characteristics (e.g., sex, age and BMI), LBP clinical features (e.g., pain intensity, disability or psychological features) and visual balance condition (e.g., when vision is removed) on postural control of the trunk. The use of IPD in this study can increase the power to detect the true effects of measured outcomes and reduce the risk of confounding and ecological bias [30, 48].

The study will also clarify similarities and differences in the methods in the literature used to measure trunk control. This will likely reveal potential factors related to experimental setups and protocols that might explain heterogeneity between studies. Results also could improve quality and reporting of studies of postural control of the trunk. An further advantage in conducting this IPD study is to connect all international collaborative researchers who are interested in the field of postural control of the trunk in individuals with or without LBP. This will provide the perfect opportunity to identify gaps in the literature and a strong platform to compare results of IPD meta-analysis with the literature.

Our study, has some potential limitations and disadvantages. First, it is possible that we will not be able to obtaining IPD set from all relevant studies that meet the eligibility criteria. Second, it is unlikely that all variables of interest can be evaluated as potential covariates. This because IPD meta-analysis depends on variables that available across multiple studies.

## Supporting information

S1 Checklist (DOC)
